# Development of a highly sensitive real-time one step RT-PCR combined complementary locked primer technology and conjugated minor groove binder probe

**DOI:** 10.1186/1743-422X-8-330

**Published:** 2011-06-29

**Authors:** JiYoung Hong, Byunghak Kang, Ahyoun Kim, Seoyeon Hwang, Jinhee Ahn, Sunhwa Lee, Jonghyen Kim, Jae-Hak Park, Doo-Sung Cheon

**Affiliations:** 1 Division of Enteric and Hepatitis Viruses, Center for Infectious Diseases, National Institute of Health, Korea Center for Disease Control and Prevention, Chungcheongbuk-do, 363-951, Korea; 2 Department of Pediatrics, Catholic University College of Medicine, Suwon, 445-744, Korea; 3 Department of Laboratory Animal Medicine, College of Veterinary Medicine, Seoul National University, Seoul, 151-742, Korea; 4 Department of Biology, College of Sciences, Kyung Hee University, Seoul, 130-701, Korea

**Keywords:** Aseptic meningitis, Real-time one step RT-PCR, CLP, MGB probe

## Abstract

**Background:**

Enterovirus (EV) infections are commonly associated with encephalitis and meningitis. Detection of enteroviral RNA in clinical specimens has been demonstrated to improve the management of patients, by ruling out other causes of disease.

**Method:**

To develop a sensitive and reliable assay for routine laboratory diagnosis, we developed a real-time one step reverse transcription polymerase chain reaction (RT-PCR) assay with minor groove binder probes and primers modified with complementary locked primer technology (TMC-PCR). We checked the sensitivity of the developed assay by comparing it to a previously published TaqMan probe real-time one-step RT-PCR (TTN-PCR) procedure using enteroviral isolates, Enterovirus Proficiency panels from Quality Control on Molecular Diagnostics (QCMD-2007), and clinical specimens from patients with suspected EV infections.

**Results:**

One hundred clinical specimens from 158 suspected viral meningitis cases were determined to be positive by the TMC-PCR assay (63.29%), whereas only 60 were found to be positive by the TTN-PCR assay (37.97%). The positive and negative agreements between the TMC-PCR and TTN-PCR assays were 100% and 59.2%, respectively.

**Conclusion:**

This data suggest that the TMC-PCR assay may be suitable for routine diagnostic screening from patient suspected EV infection.

## Background

Enteroviruses (EVs) are among the most common and important viruses infecting humans. EVs are associated with diverse clinical syndromes, ranging from mild febrile illness to severe central nervous system diseases, such as aseptic meningitis and encephalitis, potentially leading to paralysis [1,2]. Neonates and young children are at the greatest risk of developing severe, and occasionally fatal, enteroviral infections [3,4]. Serotypes of EVs have traditionally been classified into echoviruses, coxsackieviruses, groups A and B, and polioviruses [5]. Currently, EV subtypes are divided into five species (human enteroviruses [HEV] A, B, C, D, and poliovirus) with differing molecular and biological characteristics [6]. Laboratory methods used for the diagnosis of enteroviral infection have changed substantially over time [7-9]. Initially, EVs were detected exclusively by cell culture and identified by neutralization [10]. In the mid-1990s, polymerase chain reaction (PCR) methods that can detect all EVs were introduced and have supplanted cell culture in many diagnostic laboratories [11-13]. Cell culture methods for the detection of EVs are time-consuming, requiring, on average, 7-14 days for identification. Also, most coxsackievirus group A viruses do not adapt to cells as well as other EVs [14]. Although cell culture remains the gold standard for the identification of EVs in suspected patients, molecular methods such as reverse transcription PCR (RT-PCR), real-time RT-PCR, and nucleic acid sequence-based amplification offer more sensitive, specific, and rapid results [15-17]. However diagnosis by RT-PCR also has problems, including contamination from post-reaction handling and variation in results depending on laboratory staff. Several groups have described real-time RT-PCR methods for the detection of EVs in cerebrospinal fluid (CSF) [18-23].

This study was developed and validated a rapid, sensitive, and reliable real-time RT-PCR assay for the routine identification of EVs using the TaqMan minor groove binder (MGB) format combined complementary locked primer (CLP) technology [24]: the TMC-PCR assay [25,26]. After experiments to evaluate its analytical sensitivity, specificity, and reproducibility, it was used with clinical specimens from patients and the results were compared to those using a previously published TaqMan probe real-time one step RT-PCR (TTN-PCR [23]) assay.

## Methods

### Viruses and Controls

Five reference strains belonging to distinct genogroups [enterovirus 71 (EV71), coxsackievirus B2 (CVB2), echo 30 (E30), coxsackievirus A24 (CVA24), and poliovirus type 1 (P1)] were obtained from the American Type Culture Collection (ATCC) and were used to optimize TTN-PCR conditions and to evaluate analytical performance. Infectivity of viruses was assayed in microplates in serial 10-fold dilutions (from 10^-4 ^to 10^-10^) with four wells per dilution. TCID_50 _titers were calculated according to the Kärber method [27]. Enteroviral isolates including 25 serotypes (12 echovirus (E1, 3, 5-7, 9, 13, 14, 16, 1, 25 and 30), four coxsackievirus A (CVA 10, 16, 22 and 24), six coxsackievirus B (CVB 1-6), poliovirus type 1 (P1) and two new enterovirus (EV71 and 74) circulating between 1997 and 2005 in Korea were used to evaluate the reactivity of the assay to various serotypes of EVs. Enterovirus Proficiency panels from Quality Control on Molecular diagnostics (QCMD-2007) were also included to compare results from both assays.

### Clinical samples

In total, 158 clinical specimens, collected from patients with suspected viral meningitis between June and September 2008, were included for evaluation with both real-time PCR assays.

### Extraction of viral RNA

RNA was extracted from 150 μL samples with the GM Viral Nucleic Acid Extraction Kit (GreenMate Biotech Corp, Korea), according to the manufacturer's protocol using automated machines for liquid handling (Tecan, Switzerland). The GM Viral Nucleic Acid Extraction Kit uses a silica-based extraction method [28]. RNA was then recovered in 50 μL of nuclease-free water. It was used immediately or stored at -70°C.

### Primers and probe

The TMC-PCR assay for detecting EVs is based on TaqMan technology. However, to improve the sensitivity and specificity for all enteroviruses, the primers and probe described by Verstrepen *et al*. was modified [23]. The primer pair was modified with CLP technology (iNtRON Biotechnology, Korea); the forward and reverse primers had 5-mer nucleotides added in complementary sequences at their 5'-ends. To develop the TMC-PCR, the TaqMan probe was modified to an MGB-conjugated hybridization probe and was shorter than that described by Verstrepen *et al*. [23]. The TMC-PCR used two primers: NCR-cF and NCR-cR that described by Hong J *et al*. [24]. The TaqMan MGB probe, referred to TMP, was: FAM5'-CCGACTACTTTGGGTGTC-3'MGB-NFQ (positions 541-558 of the CVB2 sequence; GenBank accession number EF174469). It contains the reporter 6-carboxyfluorescein (FAM) and the non-fluorescent quencher (NFQ) dye.

### Real-time One Step RT-PCR

TMC-PCR assay was performed using an ABI Prism 7900HT sequence detection system (Applied Biosystems). Viral RNA was amplified in 25 μL reactions using RT-PCR master mix (AgPath-ID one-step RT-PCR Kit; Ambion, CA). Reactions were incubated at 45°C for 15 min, and then at 95°C for 10 min, followed by 45 cycles of 95°C for 15 s and 60°C for 40 s.

## Results

### Limits of detection and reproducibility

To determine the detection limit of the TMC-PCR and TTN-PCR assays, serial dilutions of five reference strains belonging to distinct serotypes (EV71, CVB2, E30, CVA24, and P1) were tested. The highest positive dilution of CVB2, 0.5 TCID_50_/mL, corresponded to a Ct of 35.6, whereas the lowest dilution, 1 × 10^7 ^TCID_50_/mL, corresponded to a Ct of 8.38 of P1. Each of the titrated viruses was a member of a major EV group. The minimum detectable amount of enteroviral RNA was equivalent to 0.01 TCID_50 _in CVA24. The detection limit of the end point was the same in the TMC-PCR and TTN-PCR assays (data not shown).

Reproducibility was tested by analyzing a titrated P1 virus at least three times on different days (Table 1). In the TMC-PCR assay, the Ct mean values of the 10^-2 ^(10^5 ^TCID_50_/mL) and 10^-3 ^(10^4 ^TCID_50_/mL) RNA extracts, performed in duplicate in three different runs, were 21.55 and 25.02, respectively, with intra-assay standard deviations of 0.08 and 0.07, respectively. The Ct mean values of the 10^-5 ^(100 TCID_50_/mL) and 10^-6 ^(10 TCID_50_/mL) RNA extracts, performed in duplicate in three different runs, were 30.65 and 33.95, respectively, with inter-assay standard deviations of 0.86 and 0.80, respectively.

**Table 1:** Intra- and inter-assay reproducibility of the EV MGB/CLP (TMC-PCR) and TAMRA/NonCLP real time RT-PCR (TTN-PCR)

	**Intra assay**^***a***^				**Inter assay**^***b***^			
	
	**10**^**5 **^**TCID**_**50**_**/mL**		**10**^**4 **^**TCID**_**50**_**/mL**		**100 TCID**_**50**_**/mL**		**10 TCID**_**50**_**/mL**	
	
Assay	**Mean**^***c***^	**S.D**^***d***^	**Mean**^***c***^	**S.D**^***d***^	**Mean**^***c***^	**S.D**^***d***^	**Mean**^***c***^	**S.D**^***d***^
**TTN-PCR **^*e*^	21.22	0.24	24.82	0.16	29.05	1.89	32.62	2.09
**TMC-PCR**^*f*^	21.55	0.08	25.02	0.07	30.65	0.86	33.95	0.80

^*a *^Each Ct value was determined from eight replicates within a assay

^*b *^Assays were performed three times on different days in an independent manner

^*c *^Mean of threshold cycle (Ct) value

^*d *^Standard deviation

^*e *^TAMRA/NonCLP real time RT-PCR assay

^*f *^MGB/CLP real time RT-PCR assay.

In the TTN-PCR assay, the Ct mean values of the 10^-2 ^(10^5 ^TCID_50_/mL) and 10^-3 ^(10^4 ^TCID_50_/mL) RNA extracts, performed in duplicate in three different runs, were 21.22 and 24.82, respectively, with intra-assay standard deviations of 0.24 and 0.16 respectively. The Ct mean values of the 10^-5 ^(100 TCID_50_/mL) and 10^-6 ^(10 TCID_50_/mL) RNA extracts, performed in duplicate in three different runs, were 29.05 and 32.62, respectively, with inter-assay standard deviations of 1.89 and 2.09, respectively.

### Analytical sensitivity and specificity

All clinical isolates of 25 serotypes circulating between 1997 and 2007 in Korea of EVs could be detected and there was no inconsistency between the qualitative results generated by the two assays (TMC-PCR and TTN-PCR). This confirms that the modifications to the primers and probes did not affect the detectable reaction to various EV serotypes (in other words, pan-enterovirus).

From the results for the 12 proficiency panels (EV07) from QCMD (Glasgow, Scotland), including various titers of coxsackievirus B3 (CVB3), echovirus 11 (E11), enterovirus 71 (EV71), echovirus 30 (E30), and poliovirus type 3 (P3), could be detected the genomes of EVs, including three serotypes with different detection limits, depending on the assay used (Table 2). In the each case of CVB3 (EV07-07 vs. EV07-12), E11 (EV07-03 vs. EV07-04) and E30 (EV07-08 vs. EV07-09), at a 10-fold lower viral titer than the panel, TMC-PCR assay produced a positive result, whereas TTN-PCR assay produced a negative. Parechovirus type 3 in sample EV07-02 and EV07-11 was not detected by either assay, confirming that both assays were specific to EVs.

**Table 2:** Result with Enterovirus real time quality control panels

**Panel no**.	Serotype	**TCID**_**50**_**/0.05 mL**^***a***^	**TMC-PCR**^***b***^	**TTN-PCR**^***c***^
EV07-01	Coxsackievirus B3	50	P^***d***^	P^***d***^
EV07-02	Parechovirus 3	5.6	N^***e***^	N^***e***^
EV07-03	Echovirus 11	0.13	P^***d***^	N^***e***^
EV07-04	Echovirus 11	1.3	P^***d***^	P^***d***^
EV07-05	Negative	0	N^***e***^	N^***e***^
EV07-06	Enterovirus 71	2.8	P^***d***^	P^***d***^
EV07-07	Coxsackievirus B3	5	P^***d***^	P^***d***^
EV07-08	Echovirus 30	2.5	P^***d***^	P^***d***^
EV07-09	Echovirus 30	0.25	P^***d***^	N^***e***^
EV07-10	Poliovirus type3	0.063	P^***d***^	P^***d***^
EV07-11	Parechovirus 3	0.056	N^***e***^	N^***e***^
EV07-12	Coxsackievirus B3	0.5	P^***d***^	N^***e***^

^*a *^tissue culture infectious dose per 0.05 mL

^*b *^MGB/CLP real time RT-PCR assay

^*c *^TAMRA/NonCLP real time RT-PCR assay

^*d *^positive results

^*e *^negative results.

### Comparison of TMC-PCR and TTN-PCR assays with clinical specimens

A total of 158 clinical specimens from patients diagnosed with meningitis were evaluated. Sixty were positive in both TMC-PCR and TTN-PCR assays, whereas 40 in total 158 samples were positive only in the TMC-PCR assay. The results were consistent number of negative results in both case 58/98 (Table 3). The clinical specimens consisted of 102 CSF, 48 stool, and 8 throat swab samples. To investigate assay results among different types of specimens, the TMC-PCR and TTN-PCR assays were compared using the 102 CSF samples tested; 62 and 29 samples were positive using the TMC-PCR and TTN-PCR assays, respectively. Thirty-six of the 48 stool samples were positive using the TMC-PCR assay and 29 using the TTN-PCR assay. Of the throat swab samples, 2 of 8 were positive by both assays.

**Table 3:** Comparison of results of MGB/CLP (TMC-PCR) and TAMRA/NonCLP real time RT-PCR (TTN-PCR) in different types of specimens

		**TTN-PCR **^***a***^							
		
		CSF (n = 102)		Stool (n = 48)		Throat swab (n = 8)		All specimens (n = 158)	
		
		+	-	+	-	+	-	+	-
**TMC-PCR **^***b***^	**+**	29	33	29	7	2	0	60	40
	
	-	0	40	0	12	0	6	0	58

	**Totals**	**29**	**73**	**29**	**19**	**2**	**6**	**60**	**98**

^*a *^TAMRA/NonCLP real time RT-PCR assay

^*b *^MGB/CLP real time RT-PCR assay.

In all specimens, the positive and negative agreements of the TTN-PCR assay and the TMC-PCR assay were 100% (60/60) and 59.2% (58/98), respectively. In CSF specimens, the positive and negative agreements of the TTN-PCR assay and the TMC-PCR assay were 100% (29/29) and 54.79% (40/73), respectively. In stool specimens, the positive and negative agreements of the TTN-PCR assay and the TMC-PCR assay were 100% (29/29) and 63.16% (12/19), respectively. In throat swab specimens, the positive and negative agreements of the TTN-PCR assay and the TMC-PCR assay were 100% (2/2) and 100% (6/6), respectively.

## Discussion

Several groups have developed and demonstrated molecular diagnostic methods that are highly sensitive, specific, simple, and reproducible. In the field of virology, real-time RT-PCR has become a standard diagnostic method because of its rapid turnaround time, relatively low risk of contamination, and ease of use [19,29].

This study modified a TTN-PCR published method [23] and compared it to our modified assay, TMC-PCR, for use as a routine diagnostic assay. TMC-PCR assay detected the same region of the 5'-NCR as the traditional Verstrepen amplicon. The primers used in this assay combined previously reported CLP technology [24], a probe modified at the 5'-terminal by MGB, and a 3'-terminal NFQ. These probes provided lower backgrounds and a higher signal-to-noise ratio than other hybridization probes [30]. MGB allows the design and use of shorter probes, while maintaining a high melting temperature, which may be an advantage in designing probes for small conserved regions [25,26].

The results of the modified primers and probe using the 2007 QCMD panel were compared. TMC-PCR assay detected all EVs in the panels, except parechovirus. However, the TTN-PCR assay could not detect some EVs, such as echovirus 11 and coxsackievirus B3, in the 2007 panel. These false negative results were in low viral titer samples (Table 1). To test TMC-PCR for EVs, EV71, CVB2, E30, CVA24, and P1, as example EV serotypes, were used to determine the dynamic range, to optimize real-time PCR conditions, and to evaluate analytical performance. The detection limits for EV in both assays were similar (data not shown). However, standard deviations (SDs) of the intra-assay and inter-assay reproducibility of the TMC-PCR and TTN-PCR assays were different, particularly at low viral dilutions (Table 2). Thus, TMC-PCR had better assay reproducibility.

To examine the analytical performances of the TMC-PCR and TTN-PCR assays, a prospective study on clinical samples was conducted. In 158 clinical samples from patients with aseptic meningitis, enterovirus was detected in 100 of 158 samples by TMC-PCR and 60 of 158 samples by TTN-PCR. A comparison of the TMC-PCR and TTN-PCR assays revealed 40 discrepant samples (Table 3), 33 (82.5%) of which were CSF specimens. When separately analyzed using CSF specimens, the positive and negative agreements between TMC-PCR and TTN-PCR were 100% and 54.79%, respectively. Then the GeneXpert Enterovirus Assay (GXEA; Cepheid, Sunnyvale, CA) were performed with the 40 discrepant samples to confirm the results as false negatives (data not shown). The results were matched to the results of the TMC-PCR assay. To check the false positive results, we also performed GeneXpert Enterovirus Assay (GXEA) with positive samples from TMC-PCR assay. There were no discord results between TMC-PCR and GXEA (data not shown).

The differences in viral titer were compared to the Ct mean values of CSF-positive results. The Ct mean values from TMC-PCR and TTN-PCR of positive CSF specimens were 34.84 and 32.82, respectively. The TTN-PCR was lower than TMC-PCR in terms of the absolute value of Ct, but the rate of positive TMC-PCR was higher than in the TMC-PCR assay. The results in stool specimens showed the same pattern, despite the absolute mean value of Ct differed between TMC-PCR (30.19) and TTN-PCR (28.91). Although TMC-PCR assay has better sensitivity compared with TTN-PCR assay, the Ct vale of TMC-PCR assay in certain sample is higher than that of TNC-PCR assay. The Ct value is not only factor to determine the sensitivity. In our results, TMC-PCR assay has slightly higher Ct than TTN-PCR assay but very lower standard deviation (SD) compared with TTN-PCR assay in low viral titers at inter assay (Table 1). It means that TMC-PCR assay can produce stable diagnostic result in the specimen with low viral titers such as CSF. We can confirm the high sensitivity in the low titer proficiency sample in the Table 2 based on stability induced by CLP and MGB technologies. For example in the Table 2, TMC-PCR assay produced a positive result, whereas TTN-PCR assay produced a negative result at a 10-fold lower viral titer than the panel: CVB3 (EV07-07 vs. EV07-12), E11 (EV07-03 vs. EV07-04) and E30 (EV07-08 vs. EV07-09). We also evaluate the sensitivity of assay developed in this study using clinical samples and confirm the high sensitivity of TMC-PCR assay (62/73) in the CSF sample than TTN-PCR assay (29/73). Conclusively TMC-PCR assay has slightly higher Ct value in the certain sample with high purity (culture supernatant or pure gene serial diluents), but can produce stable diagnostic results at the clinical sample with low viral titers.

The results reported that the TMC-PCR assay is a better routine diagnostic method for EV meningitis, because of the low quantities of viral RNA in CSF specimens, not only rapid and accurate results, and may be a useful molecular screening method.

## Conclusion

This study revealed that TMC-PCR assay could give reliable results and showed better reproducibility for routine laboratory diagnosis. Also technical modification of primer provided to increase sensitivity. Our data suggest that TMC-PCR assay may be more suitable to routine diagnostic assay for EV detection from clinical specimens even though TMC-PCR assay was not the novel real-time RT-PCR assay.

## List of Abbreviations

(EV): Enterovirus; (E): Echovirus; (CSF): cerebrospinal fluid; (CVA): coxsackievirus A; (CVB): coxsackievirus B.

## Competing interests

The authors declare that they have no competing interests.

## Authors' contributions

JYH, AYK, JHA and SYH performed genome analysis and cell culture. JHK and JHP drafted the manuscripts. JYH and SHL contributed to collection specimen and clinical diagnosis. JYH, BHK and DSC designed the study and critically revised the manuscript. All of the authors read and approved the final version of the manuscript.

## References

[B1] Berlin LE Rorabaugh ML Heldrich F Roberts K Doran T Modlin JF Aseptic meningitis in infants <2 years of age: diagnosis and etiology J Infect Dis 1933 168 888 892 10.1093/infdis/168.4.888 8397269

[B2] Rotbart HA Enteroviral infections of the central nervous system Clin Infect Dis 1995 20 971 981 10.1093/clinids/20.4.971 7795102

[B3] Abzug MJ Levin MJ Rotbart HA Profile of enterovirus disease in the first two weeks of life Pediatr Infect Dis J 1993 12 820 824 10.1097/00006454-199310000-00005 8284118

[B4] Modlin JF Perinatal echovirus infection: insights from a literature review of 61 cases of serious infection and 16 outbreaks in nurseries Rev Infect Dis 1986 8 918 926 10.1093/clinids/8.6.918 3541126

[B5] Oberste MS Michele SM Maher K Schnurr D Cisterna D Junttila N Uddin M Chomel JJ Lau CS Ridha W al-Busaidy S Norder H Magnius LO Pallansch MA Molecular identification and characterization of two proposed new enterovirus serotypes, EV74 and EV75 J Gen Virol 2004 85 3205 3212 10.1099/vir.0.80148-0 15483233

[B6] Tu PV Thao NT Perera D Huu TK Tien NT Thuong TC How OM Cardosa MJ McMinn PC Epidemiologic and virologic investigation of hand, foot, and mouth disease, southern Vietnam, 2005 Emerg Infect Dis 2007 13 11 1733 1741 18217559 10.3201/eid1311.070632 PMC3375788

[B7] Chapman NM Tracy S Gauntt CJ Fortmueller U Molecular detection and identification of EV using enzymatic amplification and nucleric acid hybridization J Clin Microbiol 1990 28 843 850 2161866 10.1128/jcm.28.5.843-850.1990 PMC267821

[B8] Halonen P Rocha E Hierholzer J Holloway B Hyypiä T Hurskainen P Pallansch M Detection of enterovirus and rhinoviruese in clinical specimens by PCR and liquid-phase hybridization J Clin Microbiol 1995 33 648 653 7751371 10.1128/jcm.33.3.648-653.1995 PMC228007

[B9] Watkins T Woegerbauer M Hollemann D Hufnagl P Rapid diagnosis of enterovirus infections by real-time PCR on the Light Cycler using the TaqMan format Diag Microbiol Infect Dis 2002 42 99 105 10.1016/S0732-8893(01)00330-3 11858904

[B10] Chonmaitree T Ford C Sanders C Lucia HL Comparison of cell cultures for rapid isolation of enterovirus J Clin Microbiol 1988 26 2576 80 2852672 10.1128/jcm.26.12.2576-2580.1988 PMC266950

[B11] Mamilton MS Jackson MA Abel D Clinical Utility of polymerase chain reaction testing for enteroviral meningitis Pediatr Infect Dis J 1999 18 533 537 10.1097/00006454-199906000-00011 10391184

[B12] Rotbart HA Diagnosis of enteroviral meningitis with the polymerase chain reaction J Pediatr 1990 117 85 89 10.1016/S0022-3476(05)82451-5 2164579

[B13] Tanel RE Kao SY Niemiec TM Loeffelholz MJ Holland DT Shoaf LA Stucky ER Burns JC Prospective comparison of culture vs genome detection for diagnosis of enteroviral meningitis in childhood Arch Pediatr Adolesc Med 1996 150 919 924 8790121 10.1001/archpedi.1996.02170340033006

[B14] Schmidt NJ Lennette EH Ho HH Comparative sensitivity of human fetal diploid kidney cell strains and monkey kidney cell cultures for isolation of certain human viruses Am J Clin Pathol 1965 43 297 301 14275846 10.1093/ajcp/43.4.297

[B15] Andréoletti L Blassel-Damman N Dewilde A Vallée L Cremer R Hober D Wattré P Comparison of use cerebrospinal fluid, serunm, and throat swab specimens in diagnosis of enteroviral acute neurological infection by rapid RNA detection PCR assay J Clin Microbiol 1998 36 589 591 9466785 10.1128/jcm.36.2.589-591.1998 PMC104586

[B16] Hymas WC Aldous WK Taggart EW Stevenson JB Hillyard DR Description and validation of a novel real-time RT-PCR enterovirus assay Clin Chem 2008 54 406 413 10.1373/clinchem.2007.095414 18039718

[B17] Kessler HH Santner B Rabenau H Berger A Vince A Lewinski C Weber B Pierer K Stuenzner D Marth E Doerr HW Rapid diagnosis of enterovirus infection by a new one-step reverse transcription-PCR assay J Clin Microbiol 1997 35 976 977 9157166 10.1128/jcm.35.4.976-977.1997 PMC229714

[B18] Corless CE Guiver M Borrow R Edwards-Jones V Fox AJ Kaczmarski EB Mutton KJ Development and evaluation of a real-time RT-PCR for the detection of enterovirus and parechovirus RNA in CSF and throat swab samples J Med Virol 2002 167 555 562 10.1002/jmv.10138 12116004

[B19] Dierssen U Rehren F Henke-Gendo C Harste G Heim A Rapid routine detection of enterovirus RNA in cerebrospinal fluid by a one-step real-time RT-PCR assay J Clin Virol 2008 42 58 64 10.1016/j.jcv.2007.11.016 18164234

[B20] Mohamed N Elfaitouri A Fohlman J Friman G Blomberg J A sensitive and quantitative single-tube real-time reverse transcriptase-PCR for detection of enteroviral RNA J Clin Virol 2004 30 2 150 156 10.1016/j.jcv.2003.08.016 15125871

[B21] Petitjan J Vabet A Dina J Gouarin S Freymuth F Development and evaluation of a real-time RT-PCR assay on the LightCycler for rapid detection of enterovirus in cerebrospinal fluid specimen J Clin Virol 2006 35 278 284 10.1016/j.jcv.2005.09.006 16214398

[B22] Verstrepen WA Bruynseels P Mertens AH Evaluation of a rapid real-time RT-PCR assay for detection of enterovirus RNA in cerebrospinal fluid specimens J Clin Viral 2002 25 Suppl 1 39 43 10.1016/s1386-6532(02)00032-x 12091080

[B23] Verstrepen WA Kuhn S Kockx MM van de Vyvere ME Martens AH Rapid detection of enterovirus RNA in cerebrospinal fluid specimens with a novel single-tube real-time reverse transcription-PCR assay J Clin Microbiol 2001 39 4093 4096 10.1128/JCM.39.11.4093-4096.2001 11682535 PMC88492

[B24] Hong J Kang B Kim A Hwang S Lee S Kim J Lee HY Kang SH Cheon DS Enhanced Detection of Enteroviruses in Clinical Samples by RT-PCR using Complementary Locked Primer Technology J Clin Microbiol 2010 48 615 616 10.1128/JCM.01790-09 19940056 PMC2815621

[B25] Afonina IA Reed MW Lusby E Shishkina IG Belousov YS Minor groove binder-conjugated DNA probes for quantitative DNA detection by hybridization-triggered fluorescence Biotechnique 2002 32 940 944 946-949 10.2144/02324pf01 11962616

[B26] Kutyavin IV Afonina IA Mills A Gorn VV Lukhtanov EA Belousov ES Singer MJ Walburger DK Lokhov SG Gall AA Dempcy R Reed MW Meyer RB Hedgpeth J 3'-minor groove binder-DNA probes increase sequence specificity at PCR extension temperatures Nucleic Acids Res 2000 28 655 661 10.1093/nar/28.2.655 10606668 PMC102528

[B27] Cary J Cann AJ Assays for virus infection A practical approach series, virus culture 1999 New York: Oxford University Press 83 84

[B28] Aertsens M De Cannière P Moors H Modelling of silica diffusion experiments with ^32^Si in Boom Clay J Contam Hydrol 2003 61 117 129 10.1016/S0169-7722(02)00140-7 12598099

[B29] King RL Lorch SA Cohen DM Hodinka RL Cohn KA Shah SS Routine cerebrospinal fluid enterovirus polymerase chain reaction testing reduces hospitalization and antibiotic use for infants 90 days of age or younger Pediatrics 2007 120 489 496 10.1542/peds.2007-0252 17766520

[B30] Lukhtanov EA Lokhov SG Gorn VV Podyminogin MA Mahoney W Novel DNA probes with low background and high hybridization-triggered fluorescence Nucleic Acids Res 2007 35 e30 10.1093/nar/gkl1136 17259212 PMC1865069

